# Prevalence and Sequence Analysis of Vector‐Borne Zoonotic Diseases in Stray Cats in Istanbul

**DOI:** 10.1002/vms3.70552

**Published:** 2025-08-11

**Authors:** Tuba Yazicioglu, Handan Cetinkaya

**Affiliations:** ^1^ Department of Parasitology, Faculty of Veterinary Medicine İstanbul University‐Cerrahpaşa İstanbul Türkiye

**Keywords:** cats, ehrlichia, hepatozoon, pathogens, PCR, Türkiye, vector‐borne, zoonotic

## Abstract

**Background:**

Feline vector‐borne diseases are caused by various pathogens transmitted by arthropods. Many of these infections have zoonotic importance, and cats can serve as sentinels for monitoring the health of both humans and pets. However, there is a limited research on the vector‐borne and zoonotic diseases carried by feline populations.

**Objectives:**

This study aimed to detect the prevalence of selected vector‐borne and zoonotic infections among stray cats in Istanbul, Türkiye, by molecular and phylogenetic techniques.

**Methods:**

DNA extracted from blood samples of 316 stray cats was analysed using conventional PCR assays to identify various pathogens, targeting genes 16S rRNA for *Anaplasma/Ehrlichia/Bartonella* spp., 18S rRNA for *Hepatozoon* spp., LT1 for *Leishmania* spp. and 529 bp—Repetitive element for *Toxoplasma gondii*. Phylogenetic reconstructions were conducted based on the results.

**Results:**

*Anaplasma/Ehrlichia/Bartonella* spp., *Hepatozoon* spp. and *T. gondii* prevalence were 1.8%, 3.4% and 0.3%, respectively. In addition, sequencing revealed the following prevalences: *Ehrlichia canis* (0.3%), *Hepatozoon felis* (1.5%), *Hepatozoon canis* (0.3%), *Bartonella henselae* (0.3%), *Bartonella clarridgeiae* (0.3%) and *T. gondii* (0.3%). No *Leishmania* spp. or *Anaplasma* spp. DNA was detected in any of the samples. The *E. canis* 16S rRNA gene sequence obtained in the study showed 100% homology with *E. canis* from Venezuela (human), and the *H. felis* 18S rRNA gene sequence demonstrated 99.45%–100% similarity with *H. felis* from Türkiye (*Haemaphysalis parva*).

**Conclusion:**

This study is the first to report molecular and phylogenetic findings of *E. canis* and *H. canis* in cats from Türkiye. Notably, *E. canis*, *Bartonella* spp. and *T. gondii* all have zoonotic potential, highlighting the need for surveillance within the framework of a One Health approach.

## Graphical abstract

Istanbul, the largest metropolis in Türkiye, is home to a notable population of stray cats. While cats enrich the city's culture, they can also transmit various diseases, posing diagnostic challenges for clinicians. This study aimed to detect infections in stray cats using PCR and sequencing to identify *Ehrlichia*, *Anaplasma*, *Bartonella*, *Hepatozoon*, *Leishmania* and *Toxoplasma gondii*.

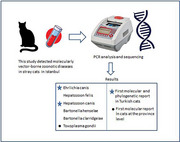

## 1 Introduction

Türkiye is known for its large population of cats, especially in Istanbul, the country's largest city. This vibrant metropolis serves as a crucial gateway to Europe. In recent years, there has been a notable increase in both companion animal ownership and stray cats, serving as reservoirs or hosts for various vector‐borne diseases. Although limited studies have focused on this topic in Istanbul, recent findings in the country indicate the emergence of diseases among the feline populations, such as *Anaplasma* spp. (Muz et al. 2021), *Hepatozoon* spp. (Koçkaya et al. 2023) and *Leishmania* spp. (Aksulu et al. 2021).

Anaplasmosis and ehrlichiosis are zoonotic diseases that occur worldwide and are transmitted to humans and animals through ticks from the *Ixodidae* family (Pennisi et al. 2017). Geographical disease distribution is linked to the competent vectors: *R. sanguineus sensu lato* and *sensu stricto* being the primary vectors for *E. canis* (Ipek et al. 2018) and *A. platys* (Snellgrove et al. 2020), respectively. In the USA, *Ixodes pacificus* and *Ixodes scapularis* transmit *A. phagocytophilum*, while *Ixodes ricinus* serves this role in Europe (Pennisi et al. 2017). *A. phagocytophilum* and *A. platys* have recently been identified in cats in Türkiye (Muz et al. 2021)—but, although *E. canis* is commonly found in dogs, it has not yet been reported in cats in Türkiye.

*Hepatozoon* spp. belong to the Apicomplexa phylum and are transmitted to vertebrates by ingesting hematophagous arthropods, primarily ticks, harbouring mature oocysts containing sporozoites. In addition, transmission can occur through predation and transplacental routes (Baneth et al. 2013). Globally, *Hepatozoon felis* has been identified as the predominant species infecting cats (Lloret et al. 2015), including those in Türkiye (Muz et al. 2021; Koçkaya et al. 2023; Önder et al. 2025). Recently, *Hepatozoon silvestris* has been detected in cats from Türkiye (Önder et al. 2025), Switzerland (Kegler et al. 2018) and Italy (Giannelli et al. 2017). *Hepatozoon canis* has been reported in cats from Europe (Giannelli et al. 2017; Diaz‐Reganon et al. 2017; Criado‐Fornelio et al. 2009) and Asia (Baneth et al. 2013; Jittapalapong et al. 2006), and it has also been found in dogs (Aktas et al. 2013), red foxes (Orkun and Nalbantoğlu 2018) and their ticks (Aktas et al. 2013; Orkun and Nalbantoğlu 2018) within Türkiye. However, no cases of *H. canis* have been reported in cats in Türkiye.

Cats are known as the primary reservoir of *Bartonella henselae*, *Bartonella clarridgeiae* and *Bartonella koehlarea* species. The most prevalent species affecting both cats and humans is *B. henselae*, which causes cat scratch disease and other potentially fatal disorders in immunocompromised individuals. *B. henselae* is typically transmitted among cats via a flea vector (*Ctenocephalides felis felis*) or in their faeces, but is commonly spread to other animals, including humans, through cat scratches. In addition, blood transfusions can pose a transmission risk (Alvarez‐Fernandez et al. 2018). In Türkiye, high rates of *B. henselae* have been molecularly diagnosed in cats (4.0%–40.0%) (Muz et al. 2021; Köseoğlu et al. 2022; Diren Sigirci and Ilgaz 2013), and *B. henselae* seropositivity was found to be statistically significantly higher (26.5%) in stray cat/dog owners (Aydin et al. 2019).

*Toxoplasma gondii* is a prevalent parasite globally, posing health risks such as abortions, neonatal complications and even fatality in humans, particularly in immunocompromised patients. Cats play a significant role in the transmission of *T. gondii* by contaminating food and water supplies with resilient oocysts in the faeces. In Türkiye, particularly Istanbul, infection rates are concerning, with seropositivity rates of 24% (Akyar 2011) among women of childbearing age and 31% (Dogan et al. 2015) in pregnant women for IgG antibodies. High seropositivity rates have also been found in cattle (24%) and sheep (25%) at local slaughterhouses. Furthermore, *T. gondii* DNA has been detected in ovine muscle samples (20%) and fermented sausage products (19%) (Ergin et al. 2009). To our knowledge, no molecular or serological screening has been conducted on cats in Istanbul for *T. gondii*, and PCR studies are limited (Karakavuk et al. 2021; Duru et al. 2017). Cat infection rates, especially among stray or free‐living populations, serve as reliable indicators of the level of *T. gondii* in the environment because infected cats can shed millions of infectious oocysts for a short period. Serological methods are commonly used for diagnosing *Toxoplasma*, but in recent years, direct detection methods, such as polymerase chain reaction (PCR), are increasingly used in both human and veterinary medicine (Dubey et al. 2020).

*Leishmania infantum* is a zoonotic disease transmitted by phlebotomine sand flies, primarily affecting dogs as the main reservoir. Although there is no evidence of the potential role of other arthropods (fleas and ticks) as vectors, *L. infantum* DNA has been detected in *Ixodes* spp., *Rhipicephalus* spp. (Pennisi et al. 2015) and *C. felis* (Persichetti et al. 2016). The prevalence of *L. infantum* is lower in cats than in dogs, but feline leishmaniosis is increasingly reported in Mediterranean countries like Italy, Spain and Türkiye, as well as in Iran and Brazil (Pennisi and Persichetti 2018). *Leishmania tropica* and *Leishmania major* are less commonly found in dogs but have been identified in stray cats in the Ege region of Türkiye (Paşa 2015; Can et al. 2016).

The diversity of these diseases and the emergence of new pathogens pose significant diagnostic challenges for clinicians. The objective of this study was to detect infections in cats from the Istanbul area using PCR and phylogenetic techniques to identify *Ehrlichia, Anaplasma, Bartonella, Hepatozoon, Leishmania* and *T. gondii*. In addition, various host factors, including age, gender and ectoparasite status, were analysed.

## 2 Materials and Methods

### 2.1 Study Area and Sample Collection

**FIGURE 1:**
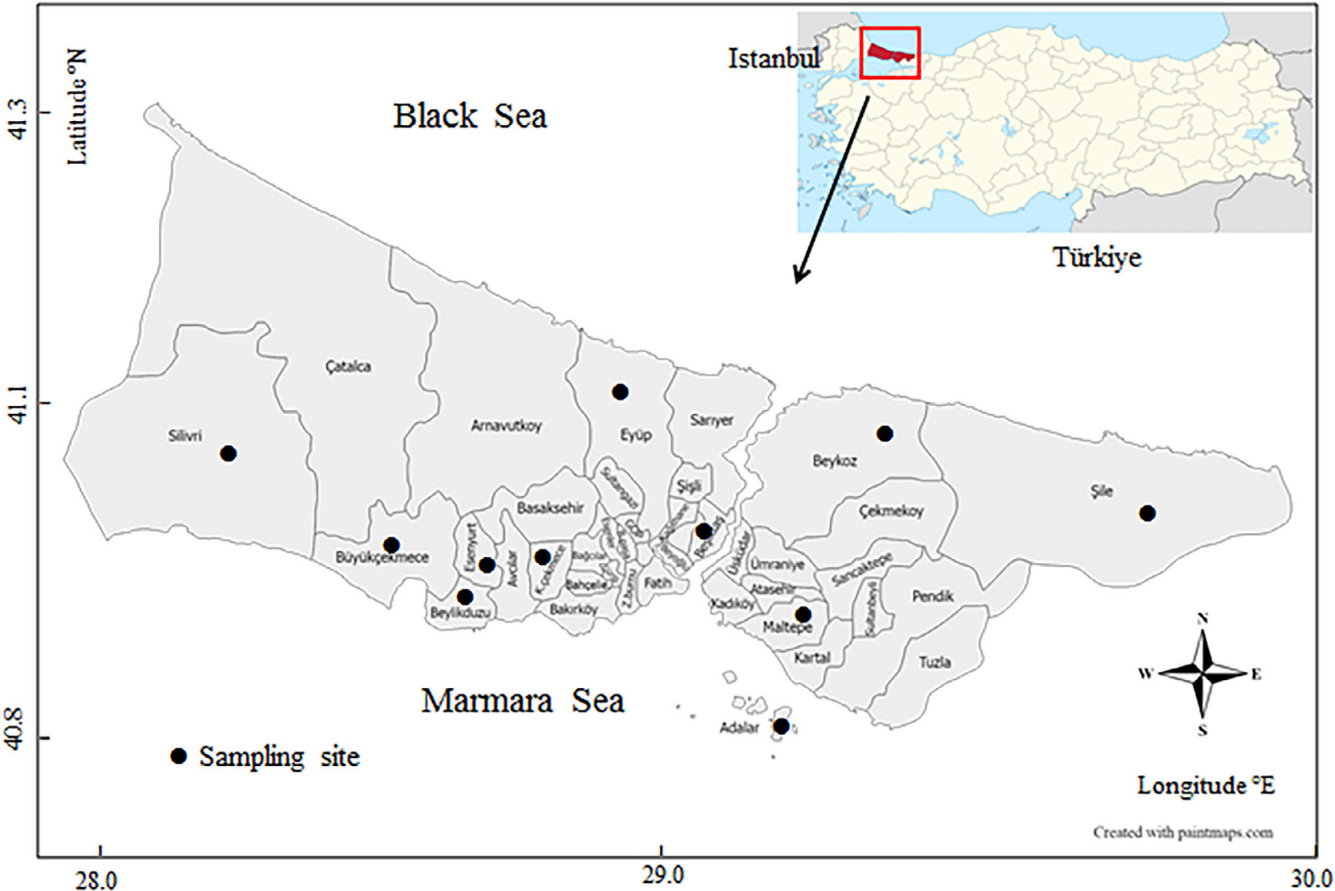
Map of stray cat sampling area.

The study sample consisted of 316 stray cats (200 ♀ and 116 ♂) from various municipal shelters (including Besiktas [*n* = 16], Beylikduzu [*n* = 40], Beykoz [*n* = 25], Buyukada [*n* = 25], Buyukcekmece [*n* = 40)] Esenyurt [*n* = 40], Eyup [*n* = 20], Kucukcekmece [*n* = 30], Maltepe [*n* = 20], Silivri [*n* = 40], and Sile [*n* = 20] districts), in the Istanbul province of Türkiye between 2018 and 2020 (Figure 1).

Peripheral blood samples were obtained from each cat by cephalic venipuncture, and the blood collected (1 mL) was placed into EDTA tubes. Subsequently, 200 µL of blood was transferred to sterile microcentrifuge tubes and stored at −20°C for molecular analyses. All experimental procedures were approved by the Istanbul University Ethics Committee (approval number: 2018/36).

### 2.2 Molecular and Phylogenetic Analysis

DNA extraction from 200 µL of blood was performed using the Extractme DNA Blood Kit (Blirt, Poland) according to the manufacturer's instructions. DNA was eluted in 100 µL of elution buffer and stored at −20°C until used. DNA concentrations were measured using a NanoDrop 2000 spectrophotometer.

The conventional PCR was carried out according to the protocols previously described for genes 16S rRNA of *Anaplasma/Ehrlichia/Bartonella* spp., 18S rRNA of *Hepatozoon spp*., LT1 of *Leishmania* spp. and 529 ‐ bp repetitive element (RE) for *T. gondii*. All primers and PCR protocols used to detect parasites are summarized in Table 1. DNA from known infected cats were used as a positive control, and nuclease‐free water was used as a negative control for all reactions. PCR reactions were carried out in a total volume of 25 µL, and for each sample, 12.5 µL master mix, 6.5 µL bi‐distilled water, 0.5 µL forward primer and 0.5 µL reverse primer and 5 µL target DNA were placed in sterile PCR tubes. The study used agarose gels prepared at 1.2% and 1.5%, considering the DNA sizes. After removing it from the microwave, 5 µL of DNA dye was added to the agarose gel (Red Safe). DNAs were loaded into the gel and run at 90–120 volts for 30–45 min. After the process was completed, the agarose gel was taken out and examined for the presence of specific bands on the UV transilluminator. Positive sample bands were photographed with a digital imaging system (iBright 1500, Invitrogen Thermofisher Imaging Systems, America).

**TABLE 1:** Primer sets, target genes, product sizes and references used in the study.

Pathogens	Primers (5′‐3′)	Gene	Product size (bp)	Reference
*Anaplasma/* *Ehrlichia/* *Bartonella* spp	F:GGAATTCAGAGTTGGATCMTGGYTCAG R:CGGGATCCCGAGTTTGCCGGGACTTCTTCT	16S rRNA	456‐476 bp	(Schouls et al. 1999)
*Hepatozoon* spp.	F:ATACATGAGCAAAATCTCAAC R:CTTATTATTCCATGCTGCAG	18S rRNA	666 bp	(Inokuma et al. 2002)
*Leishmania* spp.	F:CTTTTCTGGTCCCGCGGGTAGG R:CCACCTGGCCTATTTTACACCA	LT1	145 bp	(le Fichoux et al. 1999)
*Toxoplasma gondii*	F:CGCTGCAGGGAGGAAGACGAAAGTTG R:CGCTGCAGACACAGTGCATCTGGATT	529 bp—RE	529 bp	(Homan et al. 2000)

The PCR amplicons were sequenced by a private laboratory (BMLabosis, Ankara), and sequences were assembled using BioEdit software and compared for similarity with sequences deposited in GenBank using the basic local alignment search tool (BLAST) (http://www.ncbi.nlm.nih.gov/BLAST/). Homologous DNA sequences were aligned, and phylogenetic trees were constructed with MEGA 7.0 software (Kumar et al. 2016).

### 2.3 Statistical Data Analysis

The sample size was estimated using exact binomial 95% confidence intervals (CIs). The chi‐square test was used to analyse the results of PCR tests in relation to age, gender and ectoparasite presence of the examined cats. Values of *p* < 0.05 were considered significant.

## 3 Results

The study sample comprised 200 female and 116 male cats. Regarding age, 190 cats were one year old or younger, while 210 were older than one year. Ectoparasites (fleas) were detected on 218/316 (69%) cats. Fleas were collected from 40 of these cats, and all were identified as *C. felis felis*. All cats appeared healthy during sampling. No statistical difference was determined between age, gender, ectoparasite presence and haemopathogen PCR detection (Table 2).

**TABLE 2:** Host risk factors associated with PCR positivity for vector borne diseases in stray cats of Istanbul.

Risk factor	Variables	Number of cats (n)	PCR positive: *n* (%)	Test statistic	Degrees of freedom	*p*‐value
Age	>1 Year ≤1 Year	210 106	13 (6.1) 5 (4.7)	0.28	1	0.59
Gender	Male Female	116 200	10 (8.6) 8 (4.0)	2.91	1	0.08
Ectoparasites	Yes No	218 98	16 (7.3) 2 (2.0)	3.53	1	0.06

*p* < 0.05 indicate a significant association with PCR‐positivity.

**FIGURE 2:**
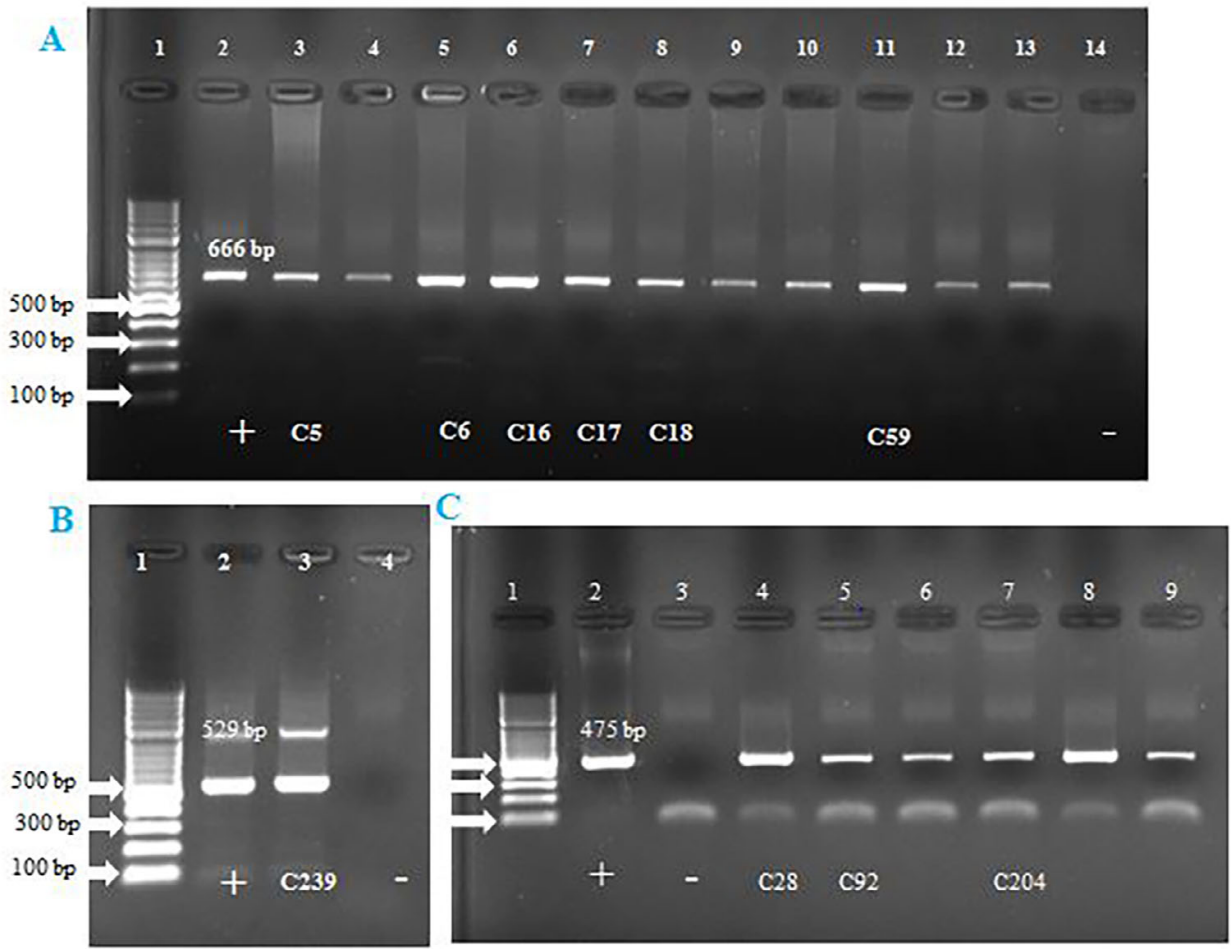
Agrose gel electrophoresis view of positive amplicons. (A) line 1; DNA ladder, line 2; positive control for *Hepatozoon* spp., line 3–13 positive samples, line 14; negative control. (B) line 1; DNA ladder, line 2; positive control for *T. gondii*, line 3; positive sample, line 4; negative control. (C) line 1; DNA ladder, line 2; positive control for *Anaplasma/Ehrlichia/Bartonella* spp., line 2; negative control, line 4–9 positive samples. The sequenced samples are indicated by their ID numbers (C5, C6, C16 etc).

Of the 316 blood samples, 18 (5.6%; 95% CI: 22.2‐35.1%) were positive for haemopathogens. The overall prevalence of *Anaplasma/Ehrlichia/Bartonella* spp. (*n* = 6, 1.8%), *Hepatozoon* spp. (*n* = 11, 3.4%), and *T. gondii* (*n* = 1, 0.3%) were identified with conventional PCR assays (Figure 2). Sequencing analysis revealed the following pathogens from the PCR‐positive amplicons: *H. felis* (*n* = 5), *H. canis* (*n* = 1), *Ehrlichia canis* (*n* = 1), *B. henselae* (*n* = 1*), B. clarridgeiae* (*n* = 1) and *T. gondii* (*n* = 1). Sequencing and BLAST analysis results are summarized in Table 3. None of the samples were positive for *Anaplasma* or *Leishmania*.

**TABLE 3:** Various host factors and homology of sequences with those of GenBank, all showing 100% query coverage.

Cat ID (Area)	Species	Gender	Age (years)	Ecto‐parasite	Accession number, similarity%, host, country	Accession number
C5 (Beylikduzu)	*H. canis*	F	>1	*C. felis*	MK957187, 99.65% Dog, Iraq	PQ608612
C6 (Esenyurt)	*H. felis*	M	>1	*C. felis*	OQ076292, 100% Cat, Turkey	PQ608613
C16 (Silivri)	*H. felis*	F	≤1	—	OQ076292, 100% Cat, Turkey	PQ657278
C17 (Silivri)	*H. felis*	M	>1	*C. felis*	OQ076292, 100% Cat, Turkey	PQ657290
C18 (Buyukada)	*H. felis*	M	>1	*C. felis*	OQ076292, 99.45% Cat, Turkey	PQ657291
C28 (Esenyurt)	*E. canis*	F	>1	*C. felis*	KJ513196, 100% Dog, Turkey	PQ608562
C59 (Beykoz)	*H. felis*	M	>1	*C. felis*	OQ076292, 100% Cat, Turkey	PQ657293
C92 (Maltepe)	*B. henselae*	M	≤1	*C. felis*	OQ165187, 100% Cat, Turkey	PQ608598
C204 (Besiktas)	*B.clarridgeiae*	M	>1	*C. felis*	AB292603, 98.40% USA	PQ657295
C239 (Eyup)	*T. gondii*	F	>1	—	PQ202301, 100% Dog, Turkey	PQ661245

**FIGURE 3:**
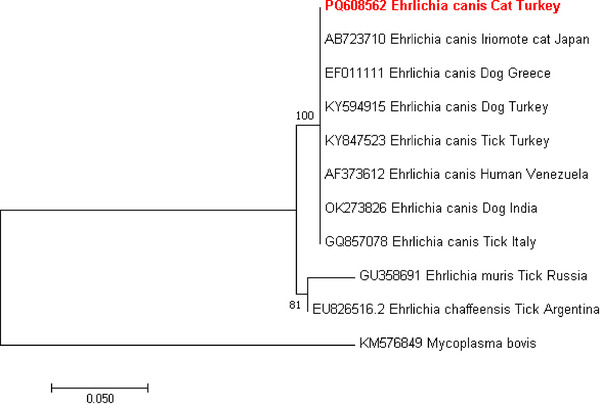
A maximum likelihood phylogram of *Ehrlichia* species was inferred from the 16S rRNA gene. The tree was constructed using MEGA version 7 software, applying the Kimura‐2 parameter model with evolutionary rates among sites (K2). To assess the confidence of the nodes and branches in the tree, bootstrap analysis with 1.000 replications was conducted. The sequences included in this study are demonstrated in boldface. The 16S rRNA gene sequence of *Mycoplasma bovis* (KM576849) was used as an outgroup.

**FIGURE 4:**
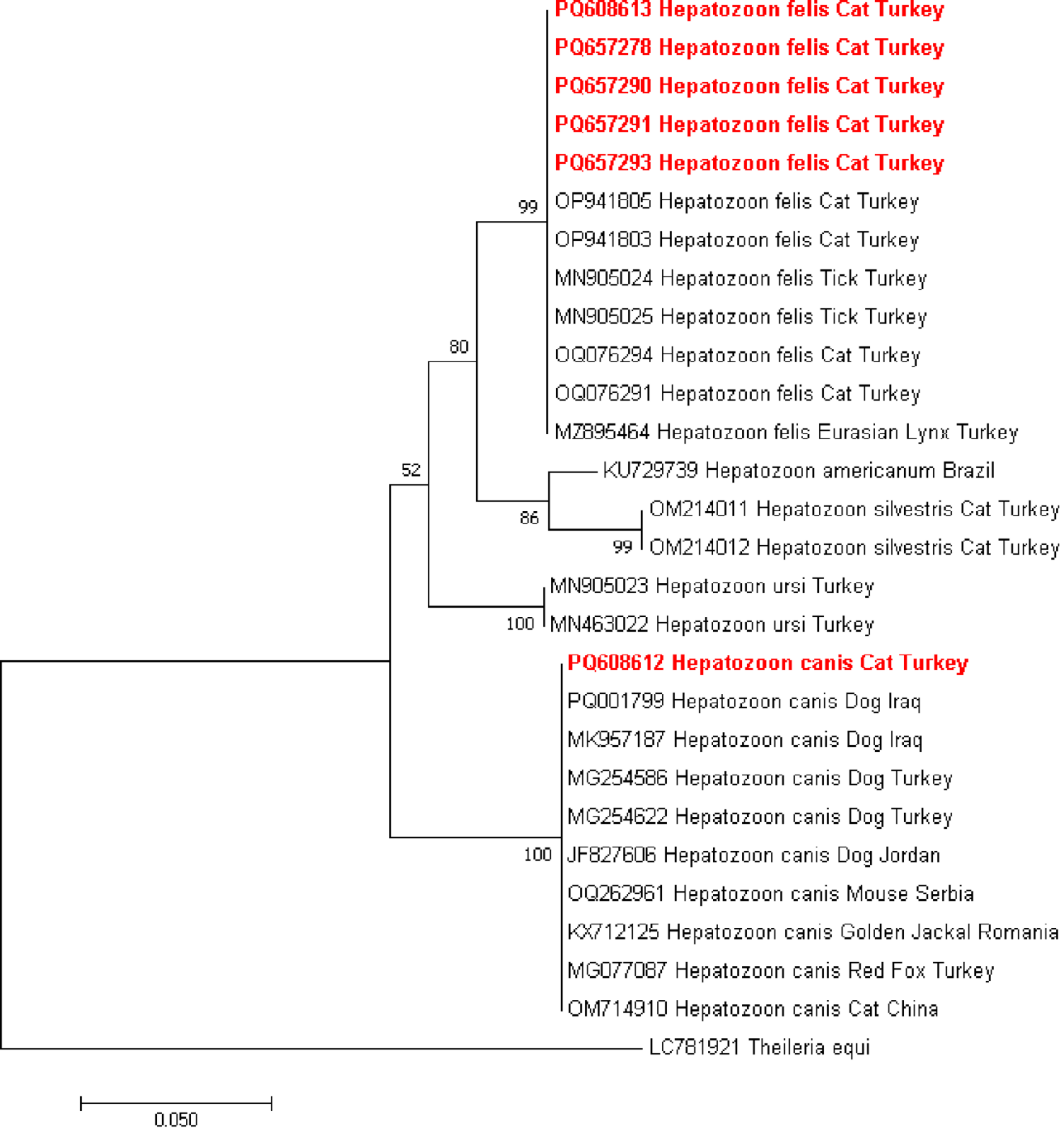
A maximum likelihood phylogram of *Hepatozoon* species was inferred from the 18S rRNA gene. The tree was constructed using MEGA version 7 software, applying the Tamura‐3 parameter model with evolutionary rates among sites (T92). To assess the confidence of the nodes and branches in the tree, bootstrap analysis with 1.000 replications was conducted. The sequences included in this study are demonstrated in boldface. The 18S rRNA gene sequence of *Theileria equi* (LC781921) was used as an outgroup.

Phylogenetic trees for *E. canis* and *Hepatozoon* spp. were constructed based on their respective genes (16S rRNA and 18S rRNA). These sequences were compared with those deposited in the NCBI GenBank database. *E. canis* 16S rRNA gene sequence obtained in the study (PQ608562) demonstrated a strongly‐supported clade with the 16S rRNA gene sequences from Venezuela (human), Italy (ticks), Turkey (dogs and ticks), Greece (dogs), Japan (Iriomote cats) and India (dogs) with a 100% homology (Figure 3). *H. felis* 18S rRNA gene sequences obtained in the study (PQ608613, PQ657278, PQ657290, PQ657291, PQ657293) established a strongly‐supported clade with the 18S rRNA gene sequences from Turkey (cats, ticks and Eurasian lynx) with a 99.45%–100% nucleotide sequence identity (Figure 4). *H. canis* 18S rRNA gene obtained in the study (PQ608612) showed a strongly‐supported clade with the *H. canis* 18S rRNA gene sequences from Turkey (dogs, red foxes), Iraq (dogs), China (cats), Romania (golden jackal), Serbia (mouse) and Jordan (dogs) with a 99.47%–99.65% nucleotide sequence identity (Figure 4).

## 4 Discussion

The first step in disease prevention is access to current data regarding disease risk. While vector‐borne diseases have been studied more extensively in dogs, both globally and within Türkiye, it is essential to recognize that cats can also act as carriers or reservoirs for many diseases (Muz et al. 2021; Önder et al. 2025; Ceylan et al. 2024). Istanbul boasts a diverse geography with a Mediterranean climate along the Marmara coast and a humid subtropical climate near the Bosporus and north. Although this research is conducted at the provincial level, we endeavoured to enhance the epidemiological reliability of our study by gathering samples from various regions of Istanbul that reflect these different climates. This diversity is important, as it may influence vector distribution. Nevertheless, studies encompassing the entire country with a larger sample size are generally more reliable when assessing the prevalence of pathogens.

The study detected several cat pathogens, including *Anaplasma/Ehrlichia/Bartonella* spp. (1.8%), *Hepatozoon* spp. (3.4%) and *T. gondii* (0.3%). Sequencing and phylogenetic analyses confirmed the presence of the dog monocytic ehrlichiosis agent *E. canis* and dog hepatozoonosis agent *H. canis* DNA in cats for the first time in Türkiye. In addition, the study identified the cat scratch disease species *B. henselae* and *B. clarridgeiae*, as well as *T. gondii*, through sequencing. Notably, *T. gondii* DNA was found in cats in Istanbul for the first time. None of the cats tested positive for *Anaplasma* spp. or *Leishmania* spp., and no statistical findings were found regarding the cats' age, gender, ectoparasite status or PCR positivity.

Ehrlichiosis is considered a rare disease in cats compared to dogs. It is not fully understood whether this rarity is due to cats' internal resistance to the pathogen (Shaw et al. 2001) or their more effective tick removal during self‐grooming (Breitschwerdt et al. 2002). Molecular detections of *E. canis* in cats have been documented in several countries, including 2.4% in Italy (Ebani et al. 2020), 3.6% in Pakistan (Abbas et al. 2023), 2.9% in Qatar (Alho et al. 2017) and 0.3% in the United States (Hegarty et al. 2015). *E. canis* is a common parasite in dogs in our country. However, only one case has been reported in which the parasite was found in a cat's blood smear and diagnosed using an immunofluorescence assay  (Albay et al. 2016). Most research on this subject has been conducted in Brazil (Braga et al. 2014, 2017; de Braga Mdo et al. 2012), with the highest prevalence reported at 9.4% (Braga et al. 2014). There is little information on the pathogenesis of rickettsial agents in cats (Pennisi et al. 2017). Surprisingly, in some studies, while PCR‐positive cats were asymptomatic (Braga et al. 2017; Kelly et al. 2017), as in dogs, severe symptoms have been described (Breitschwerdt et al. 2002). PCR is a sensitive technique for diagnosing the disease, but false negative results can occur, particularly following antibiotic treatment (Criado‐Fornelio et al. 2009). *R. sanguineus* primarily feeds on dogs, but it can also parasitize cats (Braga et al. 2017). The natural transmission cycles of this bacterium to cats are not yet fully understood. However, *R. sanguineus* may serve as a vector for *E. canis* in cats in Turkey. In the presented study, the 16S rRNA gene sequence of *E. canis* showed a strongly supported clade with the 16S rRNA gene sequences from *Rhipicephalus sanguineus* ticks from Turkey (KY847523) and Italy (GQ857078), demonstrating a 100% nucleotide sequence identity. Moreover, the sequence of *E. canis* found in cats may be zoonotically important, as it is closely related to the sequence previously identified in humans in Venezuela (AF373612) with a 100% sequence identity. *Anaplasma* spp. was not detected in the cats participating in this study. In contrast, a recent study identified *A. phagocytophilum* and *A. platys* through PCR in cats presenting to an animal hospital in Tekirdag (Muz et al. 2021). The absence of *Anaplasma* agents in the current study may be related to differences in vector distribution, the health status of the animals, or variations in the molecular techniques utilized.

*Hepatozoon* infections have been increasingly detected molecularly in cat populations in Europe (4.0%–30.0%) (Carbonara et al. 2023) and in Türkiye (2.3%–10.8%) (Muz et al. 2021; Koçkaya et al. 2023; Önder et al. 2025). In Türkiye, *H. silvestris* (Önder et al. 2025) and *H. felis* (Muz et al. 2021; Koçkaya et al. 2023) species have been diagnosed in cats, and *Hepatozoon* DNA was found in several tick species, including *R. sanguineus* (Aktas et al. 2013), *R. turanicus*, *Hyalomma marginatum*, *I. ricinus* and *Haemaphysalis parva* (Orkun et al. 2020). While the vector for feline hepatozoonosis is still unknown, it is considered that *H. parva* could potentially serve as a vector for *Hepatozoon* species in Türkiye (Orkun et al. 2020). In this study, homology was found between the four *H. felis* 18S rRNA gene sequences (PQ608613, PQ657278, PQ657290, PQ657293) from stray cats and those of *H. parva* (MN905024, MN905025) with 100% sequence identity and 100% query coverage. Likewise, this was the same homology as in the Eurasian Lynx from Türkiye (MZ895464). *H. parva* can be a competent vector for *H. felis*; however, this hypothesis requires further investigation.

*H. canis* predominantly infects domestic and wild canids, but has also been reported at low rates in cats in Europe, including 0.5% in Italy (Giannelli et al. 2017), 0.15% in Spain (Diaz‐Reganon et al. 2017) and 1.7% in France (Criado‐Fornelio et al. 2009), as in the presented study (0.3%, 1/316). *H. canis* 18S rRNA gene sequence (PQ608612) is closely related to those reported in wildlife canids and dogs from Türkiye, Iraq, Jordan and Romania, except cats from China (OM714910). Interestingly, the *H. canis* sequence from stray cats was closely related to a yellow‐necked field mouse (*Apodemus flavicollis*) from Serbia (OQ262961) with a 99.47% nucleotide identity. The yellow‐necked mouse is also found in Türkiye, especially around farmlands and buildings in rural areas. The role of this rodent is unknown; however, we consider it a potential paratenic host for *H. canis*. The phylogenetic analysis of 18S rRNA *Hepatozoon* sequences derived from stray cats in this study displays that *H. felis* and *H. canis* can infect felines in Istanbul, with *H. felis* obtaining a significantly higher prevalence.

Despite the low prevalence of *Leishmania* infection in cats, they are thought to contribute to the epidemiology and transmission of the parasite in Europe (Mancianti 2004). Molecular evidence of feline leishmaniosis has been documented at 12.6% in the Black Sea (Pekmezci et al. 2024), and 0.54% (Can et al. 2016) and 8.84% (Paşa 2015) in Ege regions of Türkiye, utilizing real‐time or nested PCR methods that target the ITS1 or Hsp70 gene regions. The failure to detect *Leishmania* in this study may be due to vector distribution and/or molecular technique variations.

*B. henselae* and *B. clarridgeiae* are frequently isolated from domestic cats, with a high reported prevalence (10%–67%) in countries with favourable temperatures and humidity favouring the flea vector, such as Italy, Spain, Portugal and Greece (Alvarez‐Fernandez et al. 2018). In Türkiye, the molecular prevalence of *B. henselae* ranges from 4% to 40%, while the prevalence of *B. clarridgeiae* varies from 0% to 9%, as reported in various studies that targeted the 16S‐23S rRNA ITS and gltA genes (Muz et al. 2021; Köseoğlu et al. 2022; Diren Sigirci and Ilgaz 2013; Aydin et al. 2019). In the present study, three of the *Anaplasma/Ehrlichia/Bartonella* positive samples could be sequenced. *B. henselae* 16s rRNA gene sequence obtained from stray cats was closely related to cats from Türkiye (OQ165187) and Brazil (ON564896) with a 100% sequence identity. Moreover, it showed 100% homology with a DNA sequence from a febrile patient (M73229) infected with human immunodeficiency virus. An assessment of the prevalence of bacteraemia based on lifestyle conditions indicates that stray cats are more likely to be bacteraemic than pet cats (Köseoğlu et al. 2022). However, our study revealed a lower prevalence in Istanbul, which may be attributed to the 16S rRNA gene providing insufficient distinction for phylogenetic analysis at the species level (Köseoğlu et al. 2022).

In recent years, genotypes II and III of *T. gondii* have been identified in domestic cats in Türkiye, which are also prevalent in Europe (Can et al. 2014). Several studies have detected these parasites in the blood of cats, showing a prevalence of 8.1% in Ankara using nested PCR (Duru et al. 2017) and 8.8% in Izmir using real‐time PCR (Karakavuk et al. 2021). Notably, this study marks the first molecular detection of *T. gondii* in cats from Istanbul, and the findings have been registered in GenBank (PQ661245). *T. gondii* 529 bp RE gene sequence obtained in the study showed 99%–100% sequence identity with sequences of cats from Sweden (U03070), Iran (LC416237) and China (KX008018). It was also closely related to other hosts, including dogs from Turkey (PQ202301), red foxes from Poland (EU165368), common wombats from Australia (KM875436) and crab‐eating foxes from Brazil (OR805035), with 100% sequence homology. Although we obtained a lower prevalence of toxoplasmosis in stray cats via PCR, detecting acutely infected cats is crucial for preventing human infections and soil contamination. The low prevalence may be due to its presence in the blood only during the acute phase, after which it migrates to the tissues. Combining serological and molecular methods can provide more effective results since stray cats may initially encounter this parasite in the early months of their lives and remain seropositive afterwards. Wider investigation is warranted since detection rates in animals and meat offered for consumption in Türkiye are alarming (Ergin et al. 2009).

## 5 Conclusion

For the first time, *E. canis* and *H. canis* were molecularly identified as cats in Türkiye. Low‐moderate levels of multiple vector‐borne pathogens were detected in the Istanbul stray cat population, and comprehensive studies are needed to ascertain the associated risks for humans.

## Author Contributions

**Tuba Yazıcıoğlu**: writing – review and editing, writing – original draft, visualization, project administration, methodology, Investigation, formal analysis, conceptualization. **Handan Çetinkaya**: writing – review and editing, supervision, conceptualization, resources.

## Ethics Statement

All experimental procedures were approved by the Istanbul University Ethics Committee (approval number: 2018/36).

## Conflicts of Interest

The authors declare no conflicts of interest.

## Peer Review

The peer review history for this article is available at https://www.webofscience.com/api/gateway/wos/peer‐review/10.1002/vms3.70552.

## Data Availability

All data supporting this study's findings are available in the article's material.
